# New findings on the male reproductive system and spermatozoa of *Aedes aegypti* (Diptera: Culicidae)

**DOI:** 10.1186/s13071-025-06808-w

**Published:** 2025-07-01

**Authors:** Henrique Barbosa da Silva, Renata Cristina Barbosa, Paulo Henrique Rezende, Dayvson Ayala-Costa, José Lino-Neto

**Affiliations:** 1 Department of General Biology, Federal University of Viçosa, Viçosa, MG 36570-900 Brazil; 2 Department of Entomology, Federal University of Viçosa, Viçosa, MG 36570-900 Brazil

**Keywords:** Spiraled testicular follicle, Sexual development, Mosquito reproduction, Microscopy

## Abstract

**Background:**

*Aedes aegypti* is one of the most important arbovirus vectors, characterized by its widespread distribution and exceptional reproductive capacity. This study reexamines the male reproductive system (MRS) of this species, focusing on its morphology throughout post-embryonic development and the structure of its spermatozoa.

**Methods:**

We analyzed the MRS of *A. aegypti* in the larval L4, pupal, and adult stages using bright-field light microscopy, fluorescent microscopy, and transmission electron microscopy techniques. Spermatozoa measurements were made using the ImageJ software.

**Results:**

In L4 larvae, the MRS was composed of the two testes, a thin deferent duct, and a pair of seminal vesicles. The MRS is fully developed in pupae and adults, with two testes, deferent ducts, seminal vesicles, accessory glands, and an ejaculatory duct. Histological sections revealed that each testis is formed by a single follicle, which appeared to spiral at all stages. In pupae and adults, the testes showed germ cells at different stages of development, while the goblet portion of the deferent duct contained cytoplasmic bodies and spermatozoa. In adults, the seminal vesicles were filled with spermatozoa soon after emergence. Secretions from accessory glands were of the apocrine type. The spermatozoa were thin and long, measuring around 335 µm in length. Ultrastructural analysis revealed a very short acrosome covering the apical nucleus, in the flagellar region, an axoneme with the 9 + 9 + ‘1’ microtubule pattern typical of mosquitoes, and two mitochondrial derivatives along the flagellum, narrowing at the terminal portion.

**Conclusions:**

Our analysis revealed a clear link between testicular development and spermatogenesis. In addition, we identified seminal vesicles at all life stages and accessory glands visible only in pupae and adults. The characterization of sperm structure and ultrastructure indicated similarities with other mosquito species. Finally, our study provided valuable information that may support research in comparative biology and reproduction.

**Graphical Abstract:**

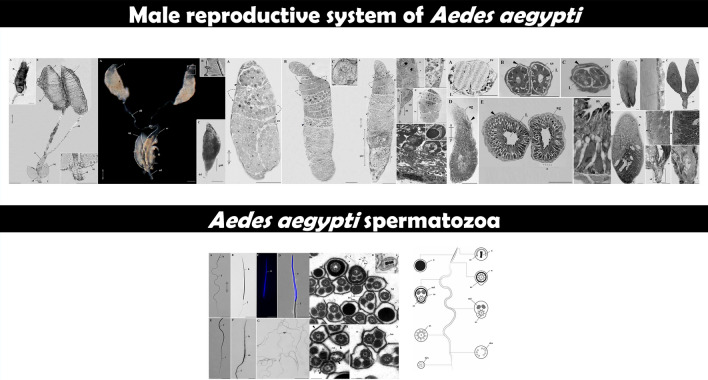

**Supplementary Information:**

The online version contains supplementary material available at 10.1186/s13071-025-06808-w.

## Background

The mosquito *Aedes aegypti* Linnaeus, 1762, is a significant vector of arboviruses responsible for diseases such as chikungunya, dengue fever, yellow fever, and Zika [1]. These diseases are of great concern because the mosquito is anthropophilic, well-adapted to urban environments, and potentially worsened by urbanization and climate change [1–4]. Half of the world’s population is at risk of dengue, with a substantial increase in cases in the last 5 years [5]. Although vaccines exist for some arboviruses, such as yellow fever and dengue, reducing the mosquito population remains the primary strategy to control these diseases [1, 2, 6, 7].

Despite not feeding on blood, male mosquitoes are essential for maintaining the mosquito population, making research into their biology crucial. The male reproductive system (MRS) produces, stores, and releases spermatozoa. In insects, this system consists of a pair of testes, deferent ducts, seminal vesicles, an ejaculatory duct and aedeago, and accessory glands [8, 9]. The testes produce spermatozoa, which exhibit great diversity, aiding in identifying different taxonomic groups [10].

Data on the MRS organization in *A. aegypti* are limited, with the descriptions dating back 60 years [11–13]. These works generally provide anatomical descriptions of the MRS, while specific data on *A. aegypti* spermatozoa morphology and ultrastructure are scarce. While some researchers have already measured the sperm size in this mosquito species, these measurements are inconsistent [12, 14]. In addition, the ultrastructure of these cells is presented, but it needs to be emphasized [15].

Although information on the MRS of *A. aegypti* is limited, we recently analyzed the MRS of the species *Lutzia bigoti* (Diptera: Culicidae) Bellardi, 1862, revealing that it is formed by a pair of testicles, deferent ducts, seminal vesicles, a pair of accessory glands, and an ejaculatory duct. Histological analysis showed that each testis is composed of a single spiral follicle [16]. Illustrations of the testicles of *A. aegypti* showed that it has a spiral appearance [12]. So, we hypothesize that this organization is a distinctive and functional characteristic of the testis, since the relationship between testicular development and sexual maturation is already known [17, 18]. Our assumption is that the spiraling of the testicular follicle is already present in late larvae (L4), since spermatogenesis begins at this stage in *Aedes* mosquitoes [19, 20]. In this work, we also describe the MRS throughout development, measure spermatozoa and analyze their ultrastructure.

## Methods

### Collection, dissection of mosquitoes, and whole-mount of the MRS

Larvae of the last instar (L4), pupae, and adults of *A. aegypti* (strain PPCampos, Campos dos Goytacazes) were obtained from the colony maintained at the insectary of the Department of General Biology at the Federal University of Viçosa (DBG- UFV). Mosquitoes in the insectary are kept under controlled conditions: 26 ± 3 °C, 60 ± 5% relative humidity, and a 12-h light–dark cycle. Larvae are fed turtle food (Reptolife®), and adults were provided with a 10% sucrose solution ad libitum. 

The *A. aegypti* larvae do not have external characteristics for determining sex, but males develop more quickly [21]. Considering this data, we collected the first larvae to reach the L4 stage of an oviposition and dissected them. We cut the abdomen of these larvae with micro-scissors, and based on the illustrations from Christophers’ 1960 [12], we performed the larval screening. Supplementary Fig. S1 shows a freshly dissected ovary. To describe the testicular anatomy, numerous larvae (L4) were dissected, and the testes were examined in whole-mount under a light microscope. For light microscopy, ten male larvae were fixed in 2.5% glutaraldehyde solution (Electron Microscopy Sciences®) in 0.1 M sodium cacodylate buffer (for at least 2 h at ambient temperature).

An initial screening was conducted to collect male pupae, as smaller pupae in *A. aegypti* are usually males [22, 23]. Ten of these pupae (at least 24 h old) were dissected, and the reproductive system was examined under a stereomicroscope to confirm it as the MRS. Some samples were prepared for whole-mount photography, and then all were fixed in 2.5% glutaraldehyde solution in 0.1 M sodium cacodylate buffer (for at least 2 h at ambient temperature).

For the collection of adult males, pupae identified as males were transferred to individual cages. Upon emergence, the sex was confirmed by the antennae, which are plumose in males [12]. These were kept in female-free cages, ensuring that only virgin individuals were used. In total, 5 newly emerged males and 40 sexually mature males (from 2 or 5 days of age, with five males at 21 days) were selected at this stage. Some of these MRS were mounted for photography. The MRS of these males were dissected and fixed as described for larvae and pupae.

Immature stages were anesthetized on ice, and adults were anesthetized with CO_2_ before their reproductive systems were dissected in 0.1 M sodium phosphate buffer (PBS), pH 7.2 solution (0.1 M NaCl, 20 mM KH_2_PO_4_, and 20 mM Na_2_HP_4_). For whole-mounts, the MRS was placed on a histological slide with a drop of PBS and covered with coverslips. Photographs were taken using an Olympus CX31® optical microscope and Zeis Primo Star® stereomicroscope equipped with a Rebel Canon T7^+^Electro-Optical System (EOS)® camera.

### Measurements of the spermatozoa

To obtain the spermatozoa, the content of the seminal vesicles from 17 adult *A. aegypti* were individually spread on histological slides under drops of PBS. Ten slides were stained with Giemsa (Merck®) (1:15 in distilled water) for 20 min, rinsed with running water, and air dried at room temperature. In total, 370 spermatozoa were analyzed, with measurements of total length (head + tail) and the thin distal portion of the tail, using an Olympus BX60® photomicroscope equipped with an Olympus Qcolor® digital camera. To highlight the nuclei, four slides were incubated in 0.2 g/L DAPI solution (4.6-diamino-2-phenylindole) (Sigma-Aldrich®), washed with running water, and 60 nuclei were photographed using the same microscope, equipped with a BP360-370 nm excitation filter. Some spermatozoa had their nuclei photographed using both fluorescent and bright field microscopy for image overlay. In addition, three slides were stained with 1% eosin (Dinâmica®) for 1 min, washed, stained with crystal violet (Dinâmica®), and washed again. The nucleus–flagellum transition region of 45 spermatozoa was photographed using the same setup. All measurements were performed using the ImageJ® software (https://imagej.nih.gov/ij/) [24].

The seminal vesicles of five newly emerged adult mosquitoes were dissected, and individual slides of their contents were prepared as previously described. At this stage, we examined whether the newly emerged mosquito already possessed spermatozoa in its seminal vesicles. Analysis and photography were performed using an Olympus CX31® microscope with a Canon EOS Rebel T7^+^® digital camera.

### Light microscopy

The larvae, pupae, and adult *A. aegypti* samples underwent a procedure where they were first rinsed in distilled water and then treated with 1% osmium tetroxide solution (Sigma Aldrich®) for 1 h. Following this, they were dehydrated using a series of increasing concentrations of alcohol (30%, 50%, 70%, 90%, and three times at 100%) for 10 min each. Subsequently, the samples were pre-treated with three different solutions of Historesin (Leica®) and alcohol (at 1:2, 1:1, and 2:1) for 1 h in each mixture. Complete infiltration was achieved by incubating the samples in pure Historesin overnight. Afterward, the samples were transferred to silicone molds containing Historesin with a catalyst added at a ratio of 15:1 and polymerized in an oven at 60 °C for 48 h.

Sections with a thickness of 1.0 µm were obtained using a Leica RM 2255® microtome. These sections were stained for 1 min with 1% toluidine blue (Synth®) in 0.5% sodium borate. Analysis and photography were performed using an Olympus CX3® microscope with a Canon EOS Rebel T7^+^® digital camera. Multiple frames were captured using either a 40× or 100× objective, and these images were later combined into panoramic views using the Photomerge tool in Adobe Photoshop 2022 Software®.

### Transmission electron microscopy

Testes and seminal vesicles from adult mosquitoes previously fixed in a glutaraldehyde solution were used in this stage. The material was washed in distilled water and then, shielded from light and at low temperature (2–3 °C), post-fixed for 2 h in 1% osmium tetroxide. Subsequently, the material was washed again in distilled water and dehydrated in ethanol of increasing concentrations: 30%, 50%, 70%, 90%, and three times in 100% (10 min at each concentration). Dehydration continued using 100% ethanol and pure acetone in increasing concentrations (2:1, 1:1, and 1:2) and pure acetone (10 min at each concentration). The material was infiltrated with Epon resin (Electron Microscopy Sciences®) overnight. The samples were then transferred to silicone molds and placed in an oven at 60 °C until complete polymerization.

Ultrathin sections (60–70 nm) were obtained using the RMC Boeckeler® ultramicrotome with a diamond knife (Diatome®). The material was then collected on grids (Electron Microscopy Sciences®) and contrasted. At this stage, the grids were placed for 40 min in a 2% aqueous uranyl solution, shielded from light. The grids were washed in distilled water and placed in lead citrate (Electron Microscopy Sciences®) for 20 min. The material was washed again in distilled water. The grids were analyzed, and the sections were photographed using the Zeiss EM 109® transmission electron microscope.

## Results

### Reproductive system in whole-mounts

**Fig. 1:**
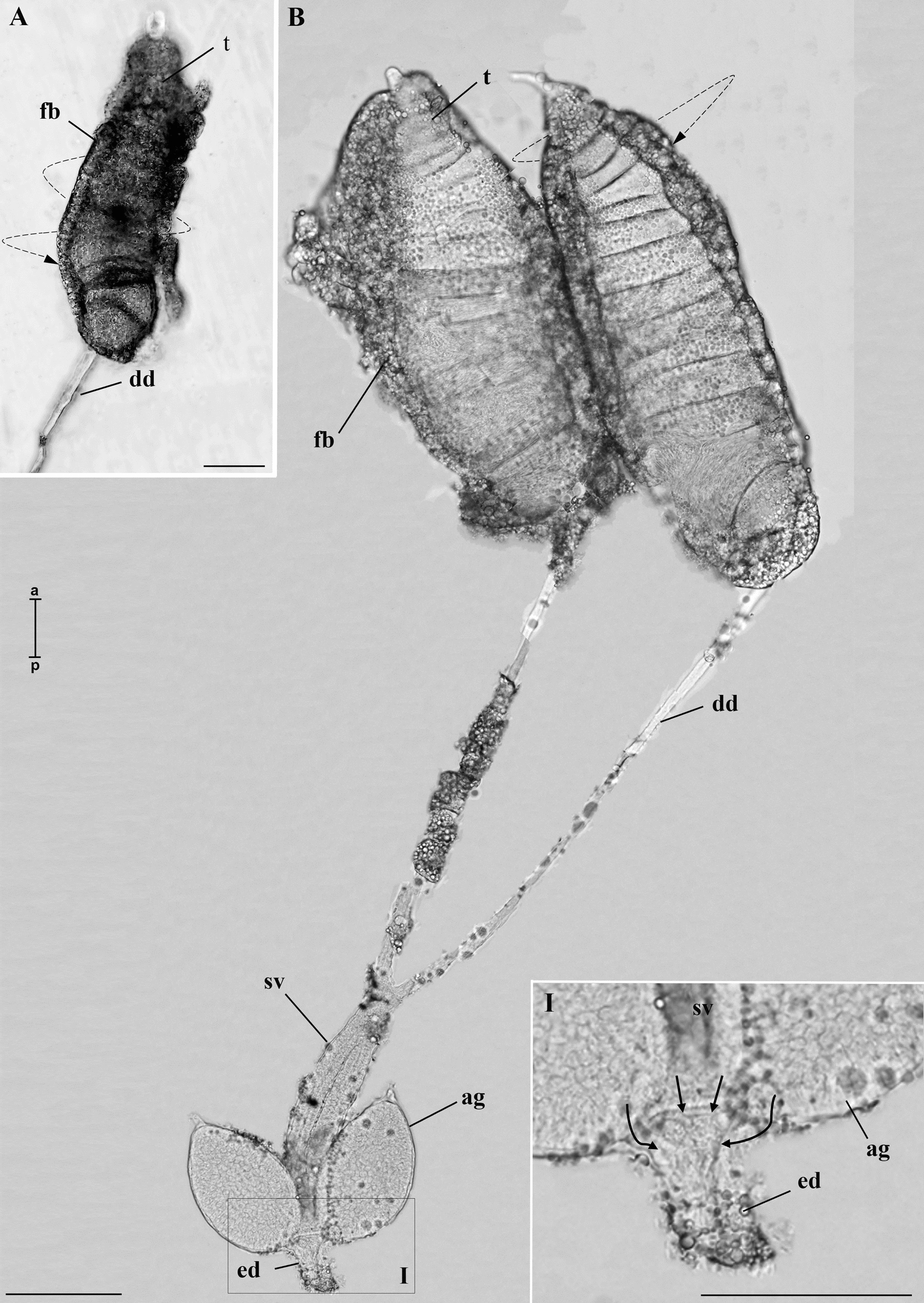
Whole-mount of the MRS of immature *A. aegypti*. **A** Testis (t) of L4 larva with a spiral shape (dotted arrow) continuous with the deferent duct (dd). **B** MRS of a pupa. The spiral shape (dotted arrow) of the testis (t) is more evident than in the L4. Each deferent duct (dd) has a dilated portion forming the seminal vesicles (sv). Note the presence of a pair of accessory glands (ag) and the ejaculatory duct (ed). Inset I shows that the ag and sv converge into the ed (arrows). In both instars, fatty bodies (fb) are noted. Scale bars: 100 µm. a, anterior region; p, posterior region

During the dissection of the L4 stage larvae, we observed the pair of testes, and each testes already displayed a slight degree of spiralization. At this stage, there is an accumulation of fat bodies surrounding them. In addition, a thin deferent duct emerges from each testis. However, no other structures were observed in the whole-mount MRS (Fig. 1A).

**Fig. 2:**
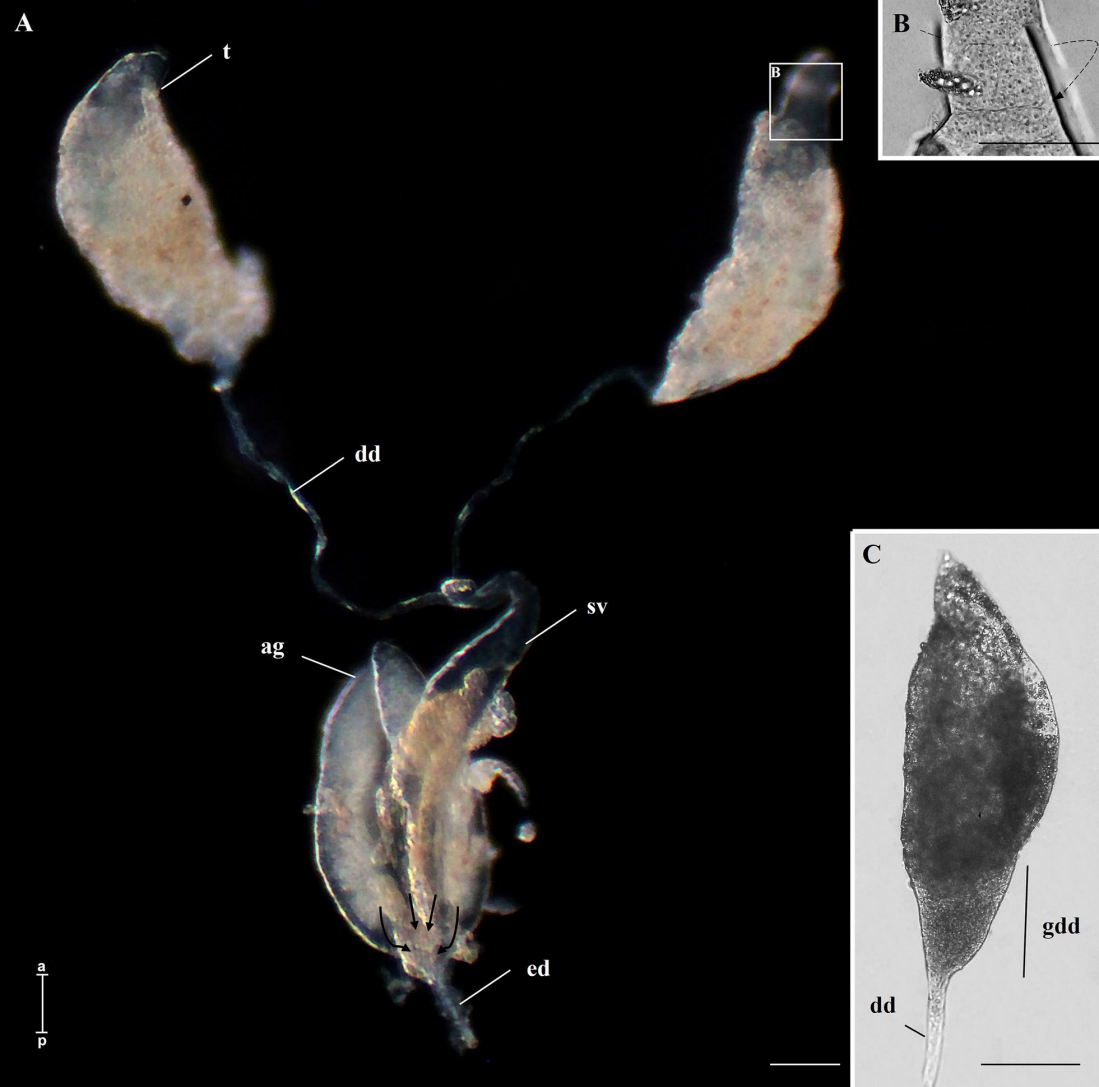
Whole-mount of MRS of *A. aegypti* adults. **A** Testis (t), deferent duct (dd), seminal vesicles (sv), accessory glands (ag), and ejaculatory duct (ed). The arrows indicate that the accessory glands and the seminal vesicles empty into the ejaculatory duct. **B** The spiral shape of the testis (dotted arrow). **C** Detail of the testis showing the goblet of the deferent duct (gdd). Scale bars: 100 µm. a, anterior region; p, posterior region

In pupae and adults the MRS was fully formed. It consisted of a pair of testes, with more evident spiralization than in L4. At this stage, a thin deferent duct that emerges from each testis has a dilation in its lower portion, forming the seminal vesicles. As observed in L4 larvae, many fat bodies were still associated with the testicular walls and the deferent ducts. Adjacent to the seminal vesicles, a pair of accessory glands can be distinguished. Glands and seminal vesicles merge at their bases, converging into the ejaculatory duct (Figs. 1B and 2A, B). In adults, it was evident that the initial portion of the deferent duct had a goblet-like shape, known as the goblet of a deferent duct. (Fig. 2C).

### Histology of MRS

#### Testes

**Fig. 3:**
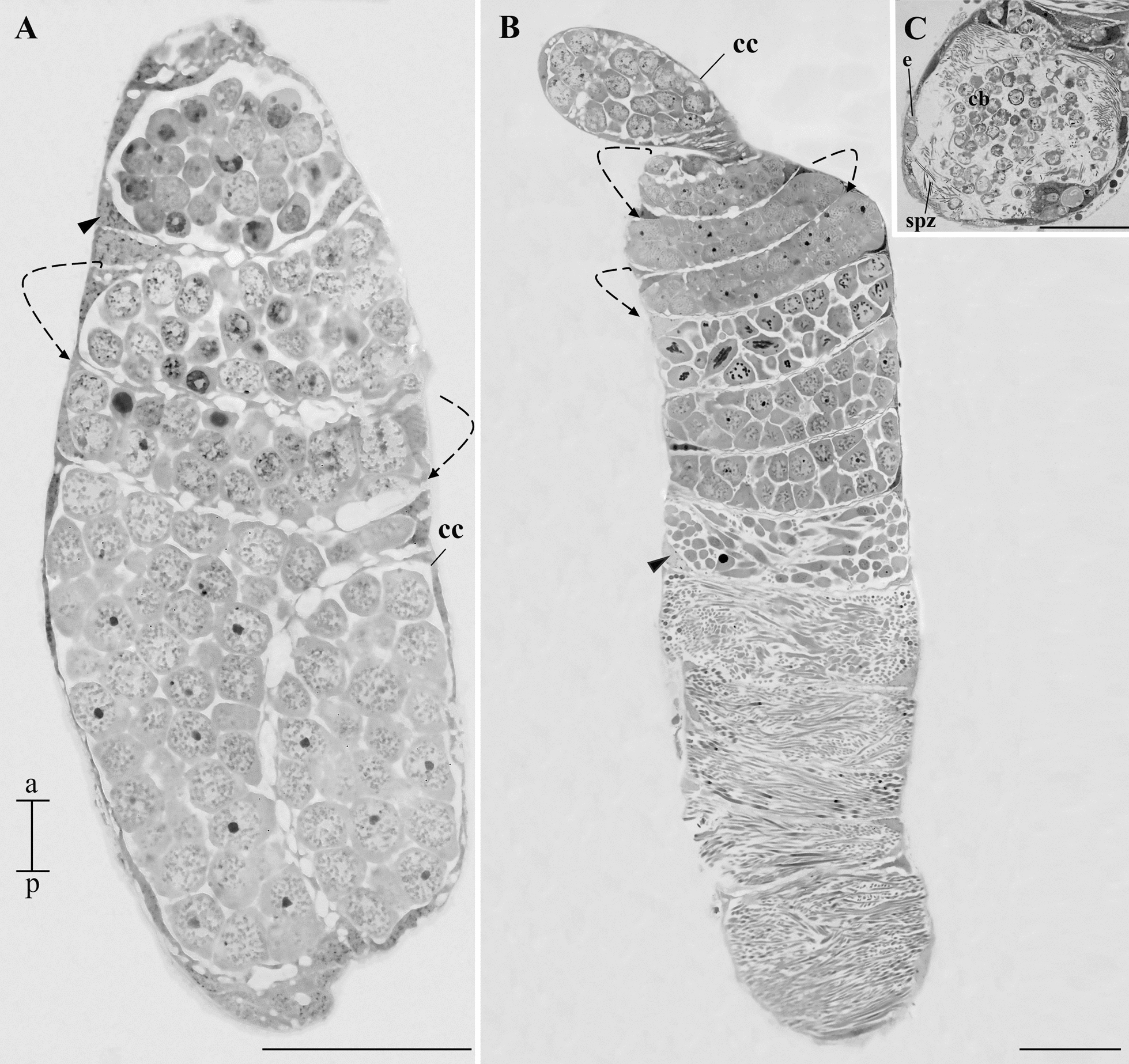
Histology of the testis of immature *A. aegypti*. **A** Testis of larva with evident spiraling of the testicular follicle (dotted arrow). Note the cytoplasm of cystic cells (cc) and their nucleus (arrowhead). The cysts contain young cells of the spermatogenic lineage. Note the presence of globular cells throughout the organ. **B** Testis of pupa. The spiraling of the testicular follicle (dotted arrow) is more pronounced. The cytoplasm of cystic cells (cc) and their nucleus (arrowhead) are also visible. Globular cells (newer in the spermatogenic lineage) are seen in the upper region of the testis while more elongated cells (older in the spermatogenic lineage) are seen in the lower regions of the testis. **C** Goblet of the deferent duct in the testis of a pupa. See the presence of an epithelium (e) in this region. Spermatozoa (spz) and cytoplasmic bodies can be seen in the lumen of the goblet. Staining: toluidine blue. Scale bar: 50 µm. a, anterior region; p, posterior region

**Fig. 4:**
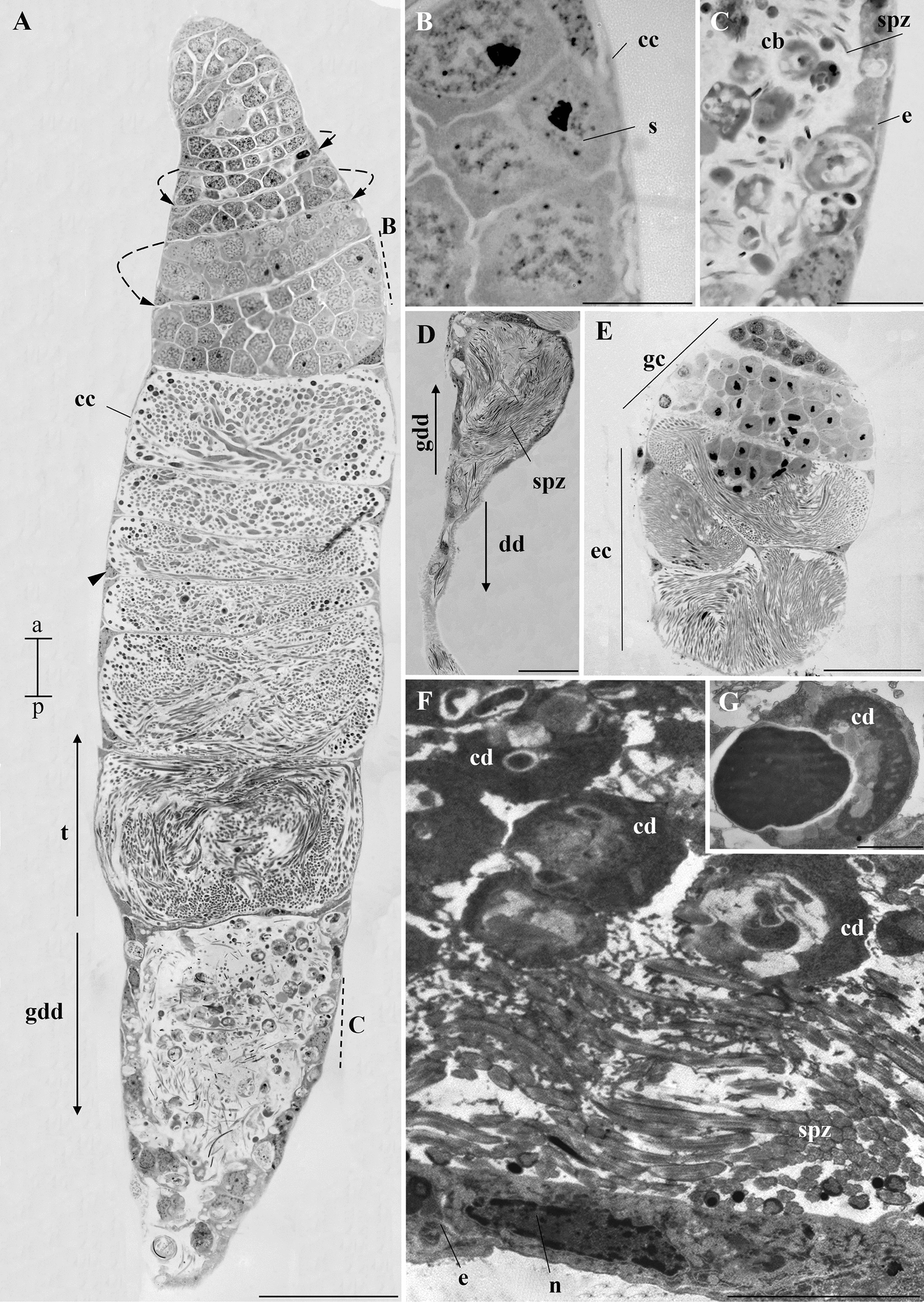
Histology and ultrastructure of the testis of adult *A. aegypti*. **A** Longitudinal histological section of the testis (t) and goblet of the deferent duct (gdd), revealing the spiral aspect of the testis (dotted arrow) and the cytoplasm of cystic cells (cc) covering it, detailed in **B**. Cystic cell nuclei (arrowhead) are present throughout the testis. The gdd is located posterior to the t and is further detailed in **C**. **B** Cytoplasm of cystic cells (cc), developing sperm cell (s). **C** Details of the gdd showing epithelium (e), numerous cytoplasmic bodies (cb), and spermatozoa (spz). **D** Histological section of the goblet of the deferent duct (gdd) and the deferent duct (dd). Note the presence of numerous spermatozoa (spz) in the lumen. **E** Cross section of the testis of *A. aegypti* at 21 days of age. The dotted arrows indicate the spiral shape of the organ. Note the presence of globular cells (gc, newer in the spermatogenic lineage) and elongated cells (ec, older in the spermatogenic lineage), indicating that spermatogenesis is still occurring at this age. **F** Ultrastructure obtained by transmission electron microscopy of the goblet of the deferent duct, identified by the presence of the epithelium (e) and a lumen filled with spermatozoa (spz). n, nucleus. Note the presence of cellular debris (cd) of various shapes in **F** and **G**. Staining **A**–**E**: toluidine blue. Scale bars: **A**, **D**, **E**: 50 µm; **B**, **C**: 10 µm; **F**: 5 µm; **G**: 1 µm

Histological sections showed that the testes from L4 larvae, pupae, and adults possess a single spiraled follicle (Figs. 3 and 4A). The histological sections confirmed the slight spiralization in the L4 testes as observed in the whole-mount. Globular germ cells, probably in the early stages of spermatogenesis, were identified in larval testes (Fig. 3A).

The sections showed that the pupal testes have a more pronounced spiraling than larvae. We observed germ cells at all stages of development. Cell development occurs in an anterior–posterior direction, with globular cells occupying the anterior region of the testicles and posterior cells in the lower region (Fig. 3B). At the base of each testis, the goblet of the deferent duct can be identified, where cytoplasmic bodies and spermatozoa were observed in the lumen. In this region, a simple epithelium is present (Fig. 3C).

Adult testes exhibit an organization like that observed in the pupae, including the 21-day-old mosquitoes (Fig. 4A–E). At all life stages, each testicular cyst is surrounded by cystic cells (Figs. 3 and 4A, B). In each cyst the germ cells are at the same stage of development. Through transmission electron microscopy, we observed the goblet region of the deferent duct is lined with a simple epithelium (Figs. 4F). The lumen of this portion contains spermatozoa and cytoplasmic bodies that correspond to cellular debris with varying shapes (Figs. 4F, G).

#### Seminal vesicles and accessory glands

**Fig. 5:**
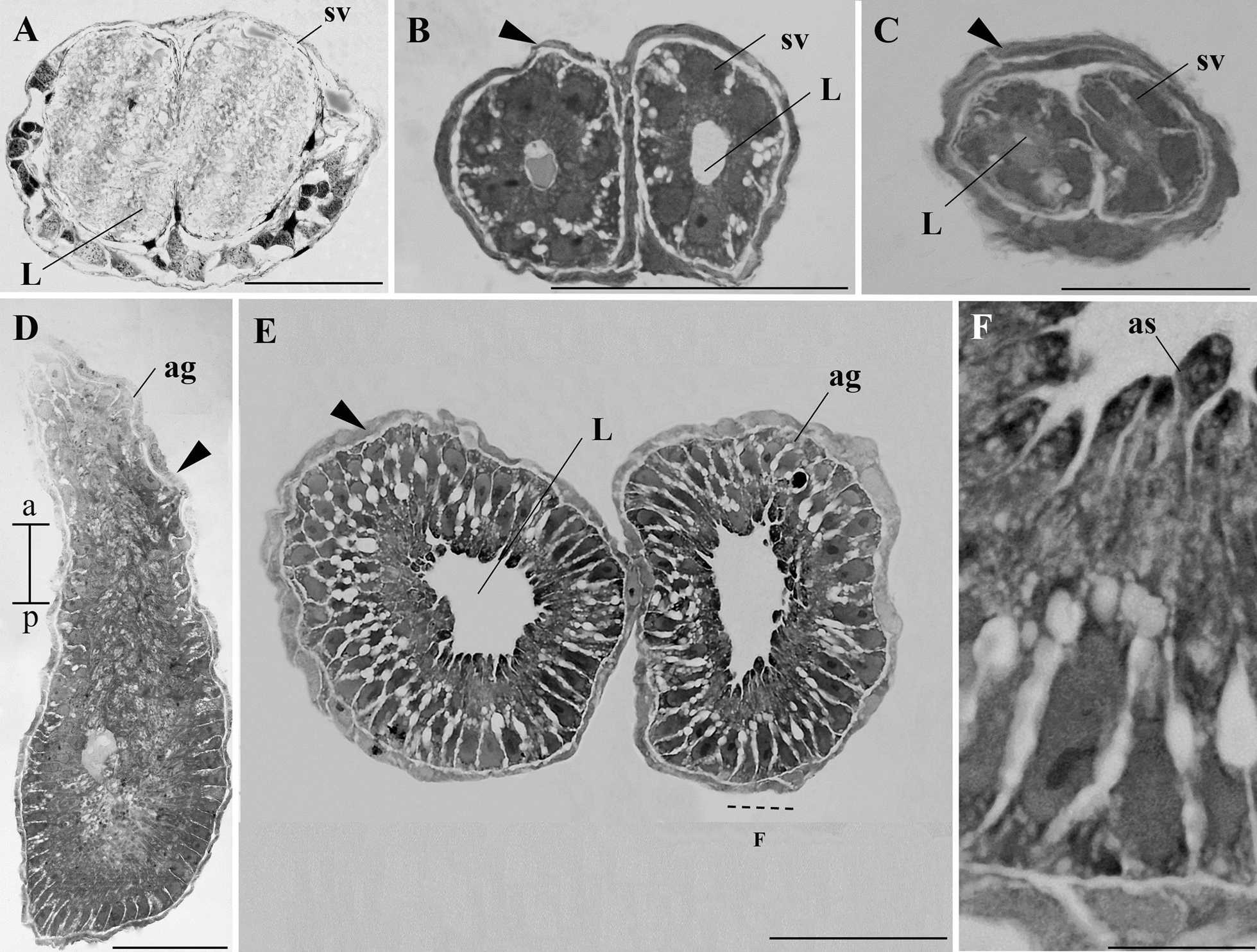
Histology of seminal vesicles and accessory glands of immature *A. aegypti*. **A** Seminal vesicle (sv) of a larva, showing a wide lumen (L). **B** Anterior portion of the pupal seminal vesicles (sv), covered by a musculature (arrowhead) surrounding each vesicle completely. **C** Posterior portion of the same sv as in **B**, with a narrower lumen (L) and no individualized muscular covering for each gland (arrowhead). **D** Longitudinal section of a pupal accessory gland (ag) surrounded by musculature (arrowhead). **E** Cross-section of two pupal accessory glands, showing the lumen (L) and the surrounding musculature (arrowhead). **F** Detail of secretory cells in the ag with apocrine secretions (as). Staining: toluidine blue. Scale bars: **A**, **B**, **D**, and **E**: 50 µm; **C**: 25 µm; **F**: 10 µm. a, anterior region; p, posterior region

**Fig. 6:**
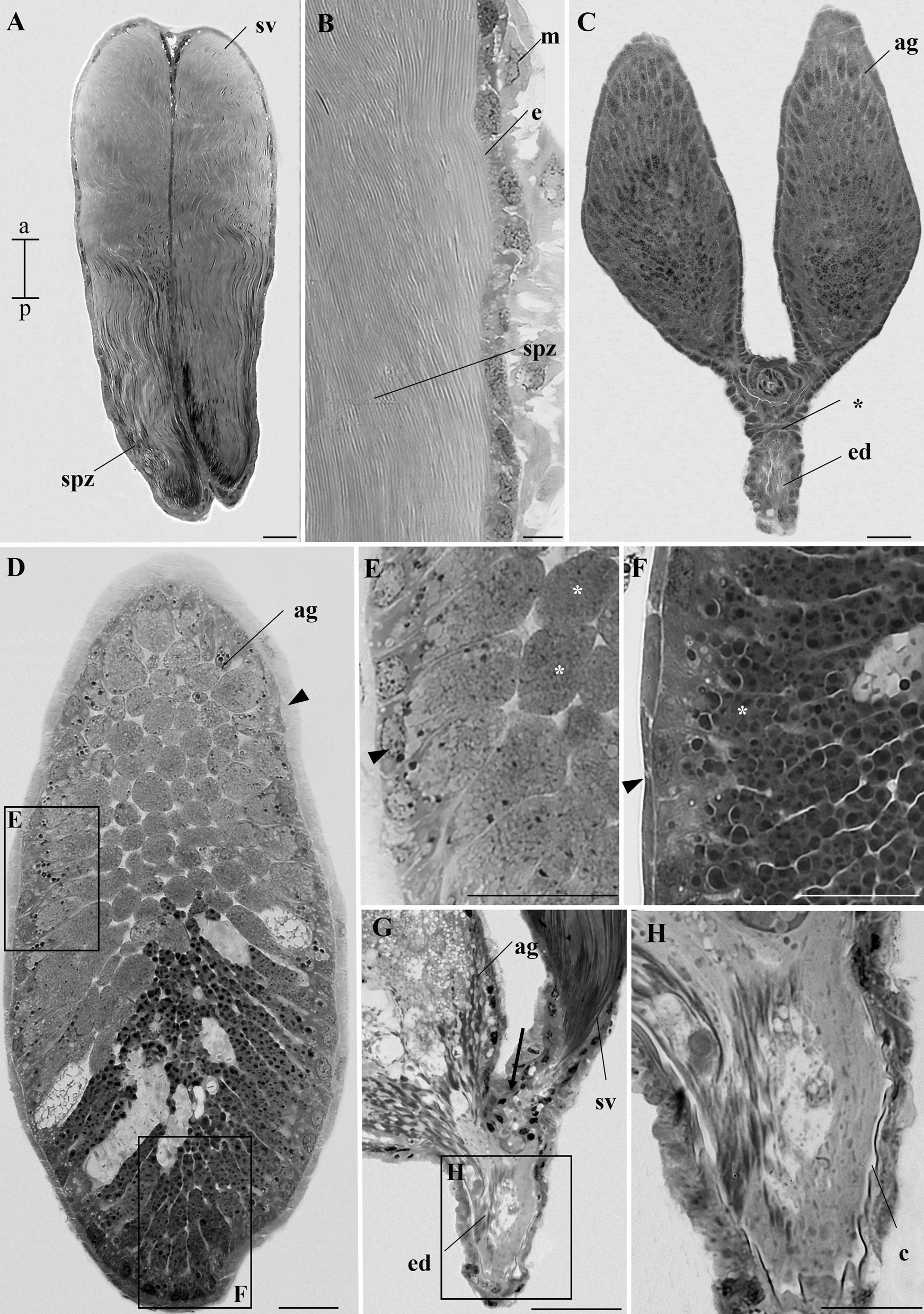
Histological sections of the seminal vesicle and accessory gland of adult *A. aegypti*. **A** Longitudinal section of two seminal vesicles (sv) filled with spermatozoa (spz). **B** Details of a seminal vesicle, showing a simple epithelium (e) and a muscular layer (m) surrounding the vesicle. Spermatozoa (spz) are visible in the lumen. **C** Junction point of two accessory glands (ag) and the ejaculatory duct (ed). **D** Longitudinal section of an accessory gland (ag) filled with apocrine secretions produced by the glandular epithelium. The ag has two distinct regions; the anterior region is stained more lightly than the posterior region. Musculature (arrowhead). **E**–**F** Longitudinal sections showing details of the secretions (*) in the anterior (**E**) and posterior (**F**) regions of the gland. **G** Junction point (arrow) of an ag with a seminal vesicle (sv), also highlighting the ejaculatory duct (ed). **H** Details of the cuticle (c) in the ejaculatory duct. Staining: toluidine blue. Scale bars: **A**–**G**: 25 µm; **H**: 10 µm. a, anterior region; p, posterior region

**Fig. 7:**
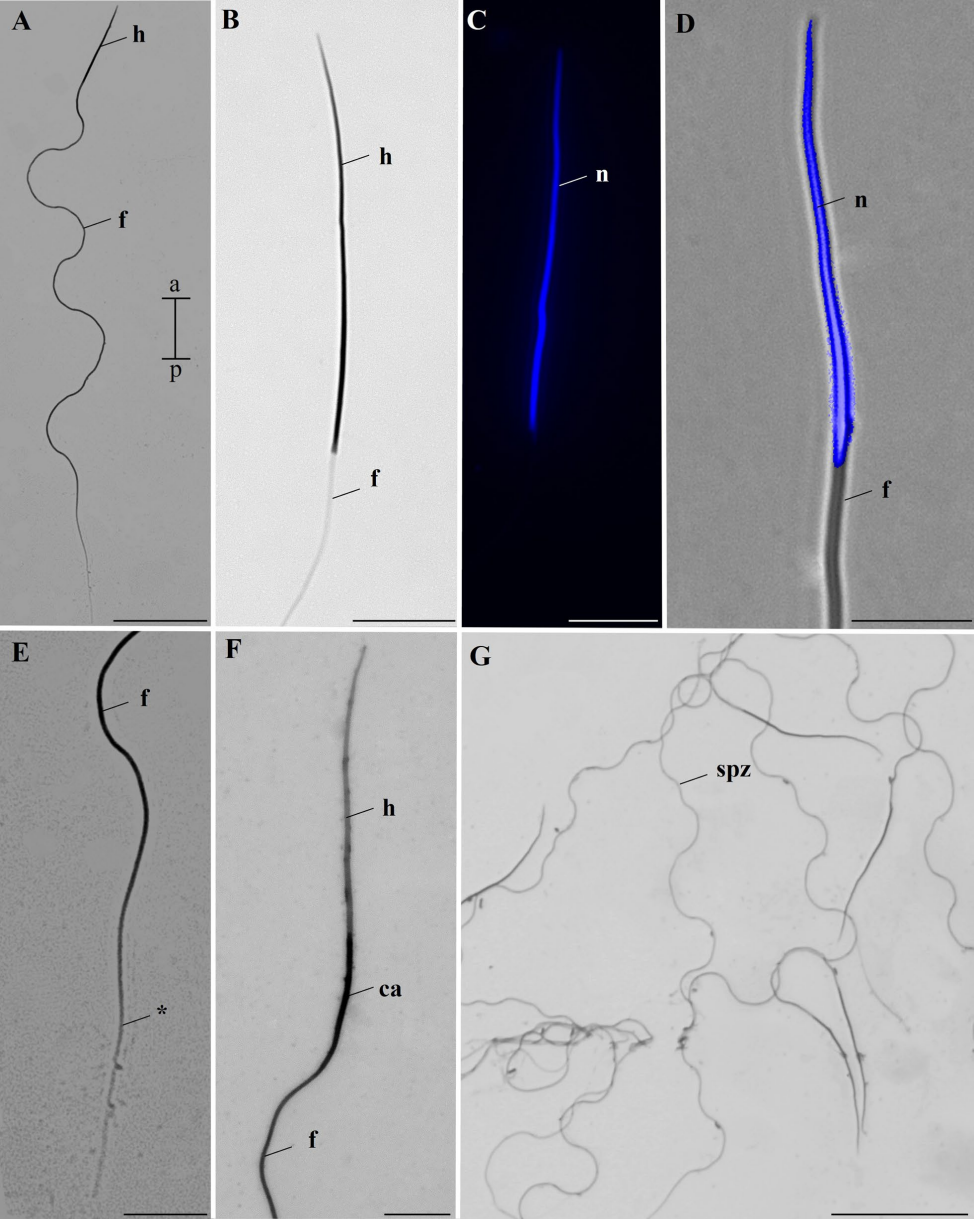
*A. aegypti* spermatozoa. **A** Overview of the spermatozoon showing the head (h) and flagellum (f). **B** Detail of the head (h) of a spermatozoon. **C** Nucleus (n) of a spermatozoon stained with DAPI. **D** DAPI and light microscopy overlay, demonstrating that the nucleus (n) occupies nearly the entire head region. f, flagellum. **E** Terminal portion (*) of the flagellum (f). **F** Spermatozoon with the centriolar adjunct (ca) highlighted. **G** Spermatozoa from a newly emerged adult. Staining: **A**, *B*, *E*, *G*: Giemsa. **F** crystal violet and eosin. Scale bars: **A** and **G**: 50 µm; **B**–**F**: 10 µm. a, anterior region; p, posterior region

Seminal vesicles were observed side-by-side at all life stages (Figs. 5A–C and 6A). Histological sections revealed that in L4 larvae, the seminal vesicles had a very thin epithelium (Fig. 5A) and contained some secretion in the lumen but lacked spermatozoa. In pupae, the seminal vesicles exhibited a simple epithelium and an empty lumen, covered by a thin muscle layer extending between the two vesicles. This muscle layer encircled both vesicles at the basal region, juxtaposing their epithelia with no muscle layer separating them in this portion (Fig. 5B, C). In adult mosquitoes, the seminal vesicles were filled with spermatozoa (Fig. 6A), present since their emergence (Fig. 7G). These vesicles had a thick epithelium composed of cuboidal cells and were enveloped by a muscle layer (Fig. 6B).

The accessory glands of pupae and adult mosquitoes featured a high epithelium producing apocrine secretions. They are lined by a muscular layer (Figs. 5D–F and 6C). The dye differentially stained the secretions of the anterior and posterior regions, with the anterior region appearing lighter than the posterior (Fig. 6D–F). The accessory glands merged at their basal portion, forming a continuous connection with the ejaculatory duct (Fig. 6G), which was internally lined by a cuticle (Fig. 6H). In L4 larvae, the accessory glands could not be identified or distinguished.

### Spermatozoa

Spermatozoa are characterized by a filiform shape, with an average length of 335.14 ± 17.58 µm (CV = 5.24%) (Fig. 7A). Observations using overlays of DAPI- and Giemsa-stained photographs of spermatozoa showed that the head of these cells is nearly entirely occupied by the nucleus, which has an average size of 38.5 ± 1.6 µm (Fig. 7B–D). The terminal portion of the flagellum is narrower than the rest, with an average length of 30.4 ± 1.8 µm (Fig. 7E). The double staining with crystal violet and Giemsa revealed the centriole adjunct, with an average length of 19.6 ± 1.4 µm. (Fig. 7F).

**Fig. 8:**
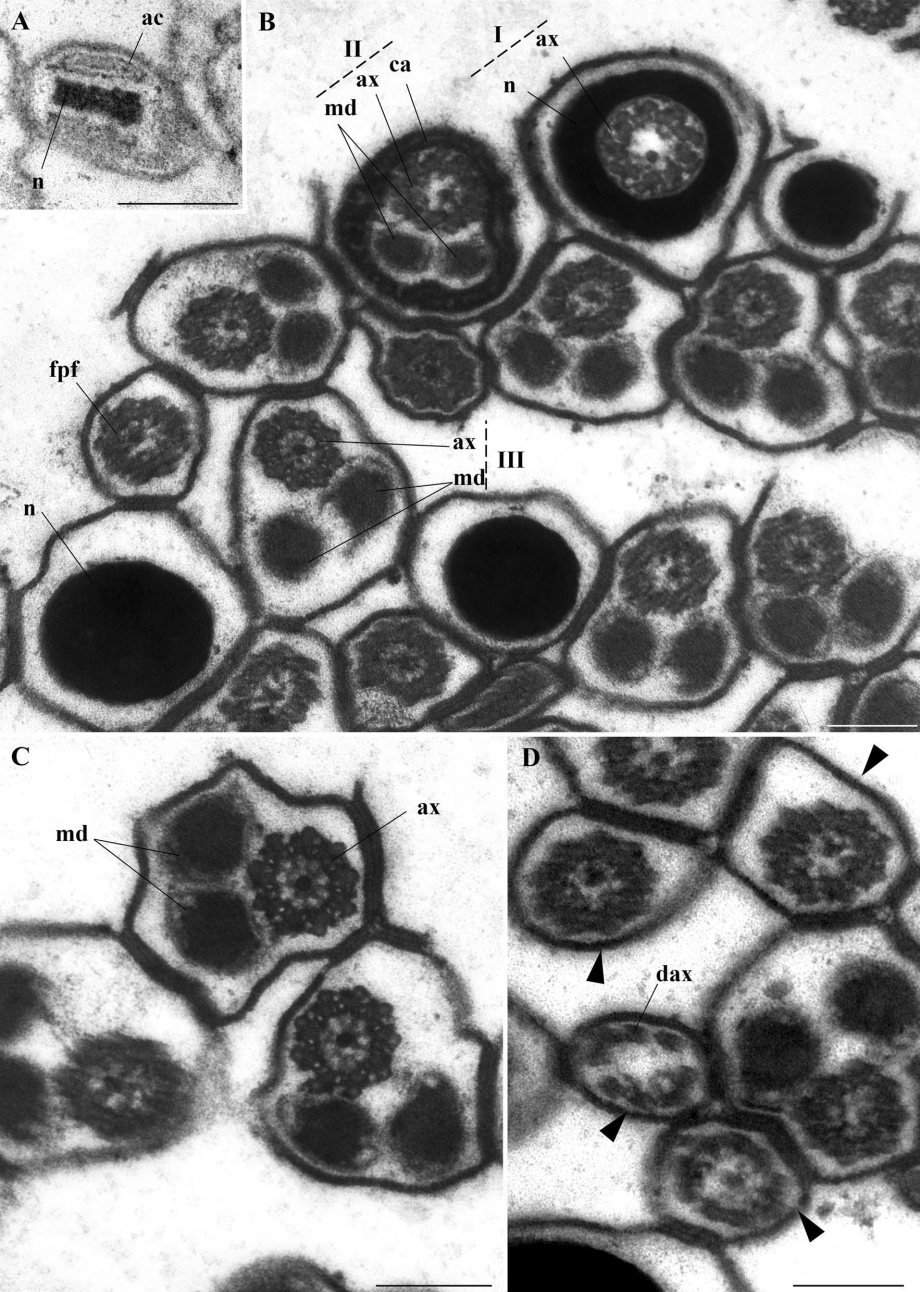
Electron micrograph of *A. aegypti* spermatozoa. **A** Anterior region of the head, showing the nucleus (n) surrounded by the acrosome (ac). **B** Several spermatozoa where the nucleus (n) is visible in I: the insertion of the axoneme (ax) into the nucleus (n); II: the centriole adjunct (ca) region containing two mitochondrial derivatives (md) and the axoneme (ax); III: the median portion of the flagellum also with two mitochondrial derivatives (md) and the axoneme (ax), and the final portion of the flagellum (fpf). **C** Median portion of the flagellum, highlighting the 9 + 9 + ‘1’ arrangement of the axoneme (ax) and the two mitochondrial derivatives (m). **D** Final portions of the sperm flagella (arrowhead), in one of them the disintegrating axoneme (dax) can be observed. Scale bar: 200 nm

**Fig. 9:**
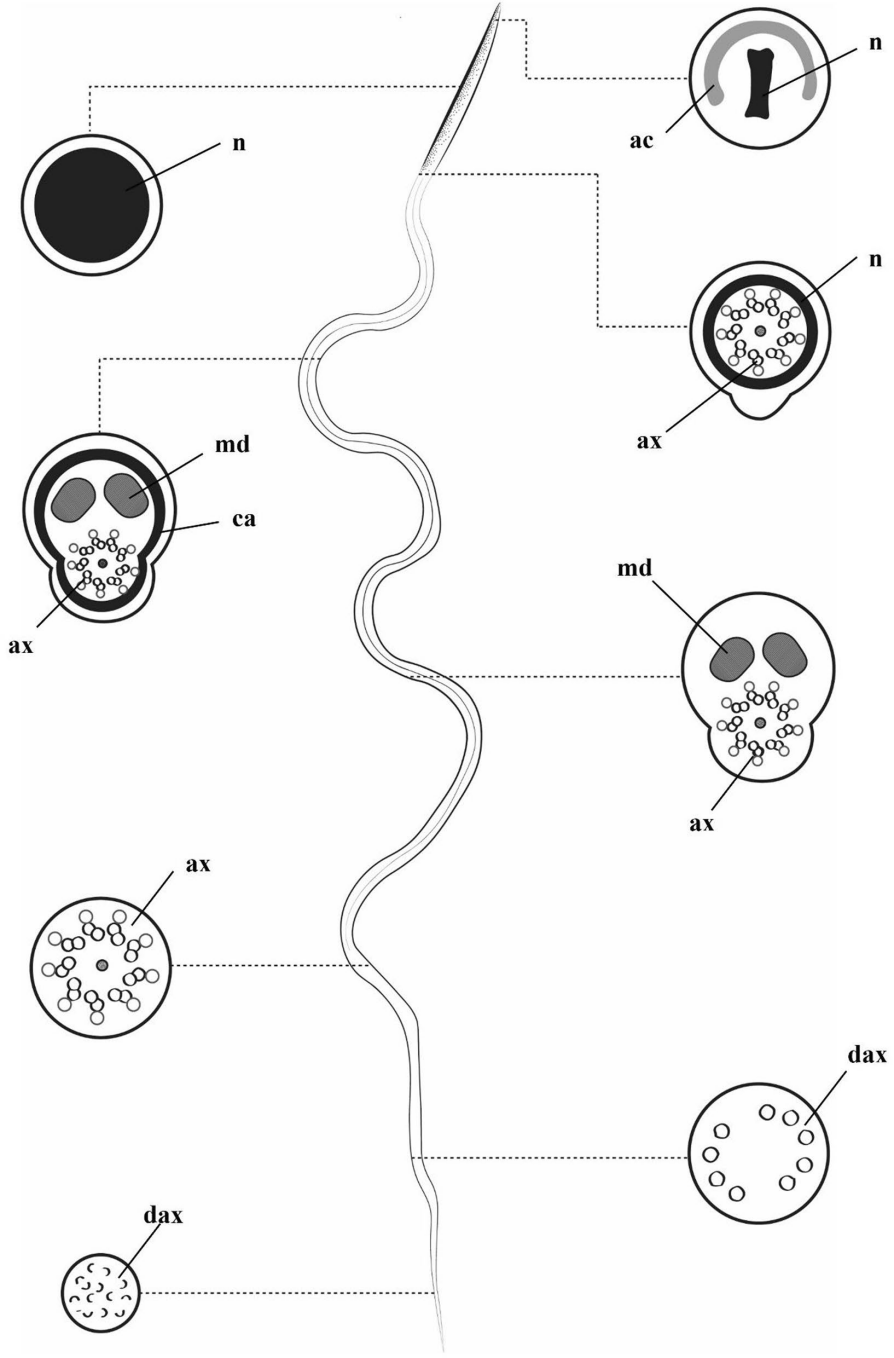
Diagram of the spermatozoa of *A. aegypti*, indicating their structures and the locations where they are found: nucleus (n), acrosome (ac), flagellum (f), axoneme (ax), centriolar adjunct (ca), mitochondrial derivatives (m), and disintegrated axoneme (dax)

The ultrastructure of the spermatozoa features a short acrosome covering the apical portion of the nucleus, which measures 0.04 µm in diameter at this region (Fig. 8A). The nucleus has condensed chromatin, and is circular in cross-section. The axoneme inserts at the nuclear base in a 9 + 9 + ‘1’ pattern (nine accessory microtubules, nine peripheral microtubule doublets, and a central element). Below this insertion, the centriole adjunct consists of granular material surrounding two mitochondrial derivatives and the axoneme (Fig. 8B, C). Further along, only the mitochondrial derivatives and axoneme remain, and the flagellar terminal portion has only the axoneme (Fig. 8D). Figure 9 shows relevant aspects of *A. aegypti* spermatozoa.

## Discussion

Our analysis revealed a strong correlation between testicular development and spermatogenesis, as the spiralization of the testes became more pronounced in pupae and adults compared with L4 larvae. This relationship is supported by the fact that spermatogenesis begins in L4 larvae and continues throughout the mosquito’s lifespan [19, 20, 25]. Therefore, we demonstrate a strong correlation between the organ’s structural development and its primary function—sperm cell production.

Compact spiraled testes were also found in *Lutzia bigoti*, another culicid species [16]. It is possible to assume that this spiral organization results in larger and elongated testes, occupying less space. This could be linked to the mosquito’s reproductive efficiency, maximizing internal space for sperm production. This idea is reinforced by the high number of sperm (about 5000) in the seminal vesicles of sexually mature mosquitoes at least 3 days old [26]. In addition, we observed that older mosquitoes continue to produce spermatozoa, allowing *A. aegypti* to be classified as synspermatogenic [27].

Each testis is continuous with the deferent duct, which in pupae and adults, has a funnel-shaped initial portion, so we refer to it as the goblet of the deferent duct [16]. Previous studies have suggested that in the testes of *A. aegypti* pupae, a mass prevents spermatozoa from leaving these organs [12]. We propose that the cytoplasmic bodies described in this study may correspond to the mass observed in the previous study but do not block sperm passage, as they are already observed in the seminal vesicles of newly emerged mosquitoes. Furthermore, these cytoplasmic bodies appear to be cellular debris, possibly originating from spermiogenesis [28].

In many insects, spermatozoa are stored in seminal vesicles [8, 9]. In *A. aegypti*, these structures are observable in histological sections as early as L4, becoming more prominent in later life stages. Although seminal vesicles have been reported in various mosquito species, including the genus *Aedes* [11, 13, 16], they have been misidentified as the ejaculatory duct in *A. aegypti* [12]. Seminal vesicles are an enlargement of the deferent ducts and store spermatozoa. In contrast, the ejaculatory duct is a shared channel that forms the aedeagus, the male intromittent organ that releases sperm cells and secretions from the accessory glands [8, 9]. Our study confirmed the presence of both structures, supporting previous findings [25], and suggesting that the Christophers (1960) [12] classification requires reconsideration in light of more recent evidence.

Accessory glands were only observed in pupae and adults. The non-observation of accessory glands in larvae could be, in fact, because of their absence or small size at this stage. Our findings align with a previous study that did not describe these glands in larvae [12]. In adults, the accessory glands are relatively larger than in pupae, likely due to the accumulation of secretory material beginning in the pupal stage and continuing through post-emergence maturation. This idea is supported by previous findings showing that the glands become empty and shrink after successive copulations, regaining their size following a period without mating [29, 30].

In pupae and adults, it was observed that the accessory glands fuse at their basal portion, a characteristic shared with other Culicinae adults [13, 16], suggesting a common trait among different genera. The ejaculatory duct receives secretions from the accessory glands and sperm from the seminal vesicles [8, 9].

In *A. aegypti*, there were conflicting reports on spermatozoa length. For instance, one study reported that spermatozoa heads measure 40–45 µm and the flagellum 200 µm [12], while another only mentioned that total length ranges from 250 to 300 µm [14]. The earliest study, conducted in the 1960s, lacks specific methodological details [12], while the second used a flat tool on a computer monitor to take measurements [14]. In our work, we used ImageJ software, which is widely used in research involving measurements and microscopy, and allows for more accurate measurements [24].

Compared with the *L. bigoti* data [16], our spermatozoa measurements suggest significant differences in sperm cell length across mosquitoes, showing remarkable variation within Culicidae. Another relevant finding is that the acrosome in *L. bigoti* is easily distinguishable in the sperm head [16], whereas in *A. aegypti*, the acrosome was visible only under transmission electron microscopy. We propose that a small acrosome may be a characteristic of the genus *Aedes*, as in *Aedes mariae* Sergent & Sergent, 1903 (Diptera: Culicidae) the acrosome measures only 0.12 µm in length [31].

The method combining eosin and crystal violet, previously demonstrated in *Apis mellifera* Linnaeus, 1758 (Hymenoptera: Apidae), can identify the acrosome, nucleus, and flagellum in spermatozoa [32, 33]. Interestingly, the same method revealed the centriole adjunct in *A. aegypti* sperm, allowing us to measure it. This structure, rich in RNA and ribonucleoproteins, is deposited beneath the nucleus during spermiogenesis and appears as an electron-dense material under transmission electron microscopy [34]. We believe this staining was possible because crystal violet, a basic dye, strongly binds to acidic components in this region.

We observed a low coefficient of variation in sperm size in *A. aegypti*. In species subject to sperm competition, it is common for more uniform sperm to be produced, with a tendency toward an “optimal phenotype” [35–42]. Polyandry, characterized by the successive mating of a female with more than one male [43], has been observed in *Aedes* females [25, 44, 45]. Thus, the low variability in gamete length may be explained by female polyandry, as males possibly produce sperm closer to the “optimal phenotype.”

Diptera has a wide variety of spermatozoa, and many differences lie in the axoneme pattern [10]. The ultrastructure of *A. aegypti* spermatozoa revealed that the axoneme is inserted at the base of the nucleus and exhibits a 9 + 9 + ‘1’ arrangement, meaning there are nine accessory microtubules, nine pairs of microtubules, and a central element that does not correspond to a true microtubule. This element is not a true microtubule, as it appears more solid than tubular [46, 47]. Although this axoneme pattern is unusual, the same arrangement has been found in other mosquito species, such as *Culex* sp., *Aedes canadensis* Theobald, 1901, and *Toxorhynchites brevipalpis* Theobald, 1901 [46–48]. Other aspects, such as the presence of the centriole adjunct and mitochondrial derivatives, were similar [46–48]. Furthermore, at the posterior end of the sperm cells, we did not observe the mitochondrial derivatives and noticed a successive loss of the axoneme elements. For this reason, under light microscopy, the posterior portion of the flagella appeared narrower.

## Conclusions

Our findings contribute to a better understanding of mosquito anatomy, particularly the morphology and post-embryonic development of the MRS in *A. aegypti*. By highlighting the importance of the spiral structure of the testes, the presence of seminal vesicles at all life stages, and the observation of accessory glands only in pupae and adults, we provide valuable insights into the mosquito’s reproductive biology. However, our research was limited by the difficulty in tracking larval stages prior to the L4 instar, as sex confirmation was unfeasible owing to the challenges in dissecting the genital apparatus. Furthermore, our focus on the structure and ultrastructure of sperm cells revealed sperm characteristics common to other mosquito species. These discoveries open new perspectives for researchers working in comparative biology or exploring male reproductive potential.

## Supplementary Information

Supplementary Material 1. Figure S1. Freshly dissected ovary from an A. aegypti L4 larva. Note the long extension (arrow) in the anterior region (a). p: posterior region. Scale bar: 100 µm.

## Data Availability

No datasets were generated or analyzed during the current study.
