# Supplementary figures and images for: New findings on the male reproductive system and spermatozoa of Aedes aegypti (Diptera: Culicidae)
Source: Parasit Vectors. 2025 Jul 1;18:246. doi: 10.1186/s13071-025-06808-w (PMC12217529; doi:10.1186/s13071-025-06808-w)

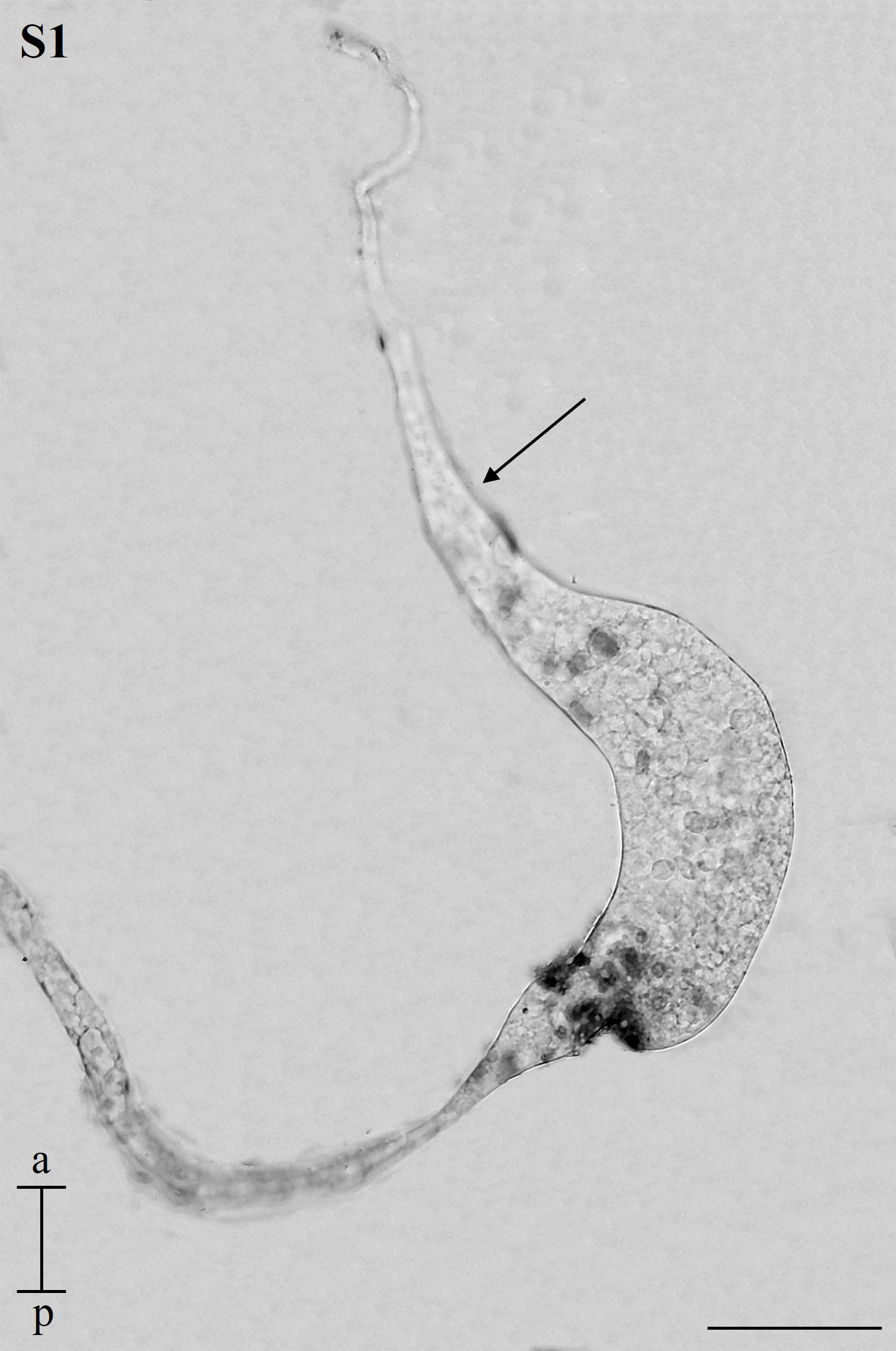

Supplement: Supplementary file 1 — Supplementary Material 1. Figure S1. Freshly dissected ovary from an A. aegypti L4 larva. Note the long extension (arrow) in the anterior region (a). p: posterior region. Scale bar: 100 µm. [file 13071_2025_6808_MOESM1_ESM.jpg]
